# Encapsulation of *Bacillus subtilis* in Electrospun Poly(3-hydroxybutyrate) Fibers Coated with Cellulose Derivatives for Sustainable Agricultural Applications

**DOI:** 10.3390/polym16192749

**Published:** 2024-09-28

**Authors:** Petya Tsekova, Nasko Nachev, Iliyana Valcheva, Donka Draganova, Mladen Naydenov, Mariya Spasova, Olya Stoilova

**Affiliations:** 1 Laboratory of Bioactive Polymers, Institute of Polymers, Bulgarian Academy of Sciences, Akad. G. Bonchev St., bl. 103A, 1113 Sofia, Bulgaria; cekovapetya@polymer.bas.bg (P.T.); nachev_n@polymer.bas.bg (N.N.); 2 Biodinamika Ltd., 4000 Plovdiv, Bulgaria; valchevailiana1@gmail.com (I.V.); donkadraganova@gmail.com (D.D.); 3 Department of Microbiology, Agricultural University, 4000 Plovdiv, Bulgaria; mladen@au-plovdiv.bg

**Keywords:** electrospinning, dip-coating, poly(3-hydroxybutyrate), cellulose derivatives, *B. subtilis*, biocontrol formulations, sustainable agriculture

## Abstract

One of the latest trends in sustainable agriculture is the use of beneficial microorganisms to stimulate plant growth and biologically control phytopathogens. *Bacillus subtilis*, a Gram-positive soil bacterium, is recognized for its valuable properties in various biotechnological and agricultural applications. This study presents, for the first time, the successful encapsulation of *B. subtilis* within electrospun poly(3-hydroxybutyrate) (PHB) fibers, which are dip-coated with cellulose derivatives. In that way, the obtained fibrous biohybrid materials actively ensure the viability of the encapsulated biocontrol agent during storage and promote its normal growth when exposed to moisture. Aqueous solutions of the cellulose derivatives—sodium carboxymethyl cellulose and 2-hydroxyethyl cellulose, were used to dip-coat the electrospun PHB fibers. The study examined the effects of the type and molecular weight of these cellulose derivatives on film formation, mechanical properties, bacterial encapsulation, and growth. Scanning electron microscopy (SEM) was utilized to observe the morphology of the biohybrid materials and the encapsulated *B. subtilis*. Additionally, ATR-FTIR spectroscopy confirmed the surface chemical composition of the biohybrid materials and verified the successful coating of PHB fibers. Mechanical testing revealed that the coating enhanced the mechanical properties of the fibrous materials and depends on the molecular weight of the used cellulose derivatives. Viability tests demonstrated that the encapsulated *B. subtilis* exhibited normal growth from the prepared materials. These findings suggest that the developed fibrous biohybrid materials hold significant promise as biocontrol formulations for plant protection and growth promotion in sustainable agriculture.

## 1. Introduction

Plant diseases are a worldwide problem that causes enormous damage to agricultural crops every year [1]. The main causes of diseases are various plant pathogens [2]. Currently, chemical pesticides are widely used to combat plant diseases. In the long term, however, their excessive application leads to soil, water, and air pollution; harm to insects, birds, and mammals; and can be dangerous to human health [3]. Therefore, the gradual and phased cessation of the use of all chemical pesticides through the mass application of so-called “eco-friendly agriculture”, should be accomplished. This necessitates the creation of safe alternatives to protect crops from pesticides and diseases. In this regard, innovative biocontrol agents can accelerate the process of reducing reliance on pesticides and thereby ensure a sustainable eco-agriculture that is safe for the environment. This will contribute to a smooth and sustainable transition to an eco-friendly society.

Due to its great potential as an alternative to chemical pesticides, biological disease control is now widely recognized and represents an important tool in integrated pest management. Biocontrol refers to the use of (micro)organisms to control plant diseases or pests [4,5]. So-called “biocontrol agents” occur widely in nature and include bacteria, fungi, viruses, yeasts, and protozoa, and can control plant diseases directly or indirectly [6,7,8,9]. The first way implies a direct antagonistic effect of the biocontrol agent on the pathogen, which can be achieved through parasitism, antibiosis, and competition for nutrients or sites of infection. In the indirect mode of disease control, the biocontrol agent induces reactions in which the plant is the mediator, allowing the plant to respond more quickly and more effectively to the next pathogen attack [10]. As a result of this biocontrol, crop yields are increased and the risk of contamination with toxic substances from widely applied chemical pesticides is reduced. Therefore, biological control products, as an alternative to chemical pesticides, target pests or pathogens by suppressing or stopping their development, but can also stimulate plants [11]. The stimulating effect is expressed in accelerating growth and increasing the resistance of plants to phytopathogens [12]. Various beneficial microorganisms (bioagents) exhibit such activity, some of which are used in biological products for plant protection and as biological soil improvers. A number of studies have shown that soil bacteria of the *Bacillus* species are promising biological agents against a number of pathogens [13,14]. Moreover, the biological control carried out by Trichoderma and Bacillus against pathogenic organisms affecting the seeds and roots of various plants is less harmful and more effective than treatment with chemical pesticides [15]. Thus, the identification of novel biocontrol agents and the creation of novel innovative biohybrid materials represent critical steps in the development of biocontrol products that can significantly accelerate the process of reducing dependence on pesticides and thus ensure a sustainable eco-agriculture safe for the environment and society.

Encapsulation of selected bioagents into targeted designed polymer carriers is a rational way to develop innovative biohybrid materials as biocontrol formulations [16]. In particular, the inclusion of beneficial microorganisms in a suitable polymer matrix not only protects them from biotic and abiotic stress, but also maintains their metabolic activity for a long period of time after their application [17]. Moreover, their controlled release is ensured, the doses and time of their application are reduced, and the negative impact of environmental factors is decreased. An advantage is also the fact that the polymer matrix, in addition to being a carrier, can also serve as a substrate for the development of biocontrol agents, i.e., it can have an active role in delivering them into the environment for plant development. In this context, natural polymers obtained from renewable sources have come to be accepted as one of the most promising biopolymers with agricultural applications [18]. Among them, poly(3-hydroxybutyrate) (PHB), a semi-crystalline microbial polyester, has the advantage of exceptional biodegradability and biocompatibility over the other thermoplastics. Moreover, PHB has been considered to possess great potential in terms of replacing petrochemical polymers such as polypropylene and polyethylene [19]. Recently, we have demonstrated the potential of electrospun PHB composite materials in agriculture for plant protection against the penetration and growth of *P. chlamydospore*—one of the main fungi causing the esca disease in grapevines [20]. In addition, for the first time, we have revealed that coating of PHB with appropriate natural polymers is an alternative approach to overcoming its brittleness and to improving its mechanical properties [21].

In this study, a promising, simple, and effective approach was developed to prepare eco-friendly biohybrid materials for use in eco-agriculture. This approach combines the favorable properties of PHB and cellulose derivatives with those of a biocontrol agent. *Bacillus subtilis* was chosen as the biocontrol agent, while different cellulose derivatives were used for dip-coating the electrospun PHB materials. The effects of the type and molecular weight of the cellulose derivatives on dynamic viscosity, film formation, mechanical properties, bacterial encapsulation, and growth were thoroughly investigated. In addition, detailed characterization of the obtained biohybrid materials, including morphological, physical, and mechanical properties, was performed. Finally, microbiological tests confirmed the potential of the PHB-based fibrous biohybrid materials as effective biocontrol formulations for plant protection and growth promotion in sustainable eco-agriculture.

## 2. Materials and Methods

### 2.1. Materials

Poly(3-hydroxybutyrate) (PHB) with an average Mw 330,000 g/mol was supplied from Biomer (Schwalbach, Germany). Sodium carboxymethyl cellulose (CMC-Na) with an average Mw ~250,000 g/mol and degree of substitution of 0.9, and 2-hydroxyethyl cellulose (HEC) with an average Mv ~90,000 g/mol (HEC-L), average Mw ~250,000 g/mol (HEC-M), and typical Mv ~1,300,000 g/mol (HEC-H) were purchased from Sigma-Aldrich (Darmstadt, Germany). Chloroform (CHCl_3_), *N*,*N*-dimethylformamide (DMF), and potato dextrose agar medium were supplied from Merck (Darmstadt, Germany). All chemicals were of analytical grade and used as received. Disposable consumables were delivered by Orange Scientific (Braine-l’Alleud, Belgium).

The *Bacillus subtilis* microorganisms were obtained from the collection of Biodinamika Ltd., Plovdiv, Bulgaria. The bacteria were grown in liquid medium, Tryptic Soy Broth (TSB) Biolife (Milan, Italy). Cultivation was carried out on a rotary shaker at 197 rpm and 28 °C, until complete sporulation occurred. The spores were harvested by centrifugation at 6000 rpm and 4 °C for 15 min, and washed twice with sterile distilled water. The final spore concentration was determined to be 1 × 10^10^/mL.

### 2.2. Obtaining the Electrospun PHB Materials

The fibrous PHB materials were produced by electrospinning a PHB solution. The solution was prepared by dissolving PHB (14% *w*/*v*) in a mixed CHCl₃/DMF (4/1 *v*/*v*) solvent at 60 °C using a reflux. The resulting solution was then loaded into a 20 mL plastic syringe fitted with a 20-gauge needle and connected to a positively charged electrode. This electrode was attached to a custom-made high-voltage power supply capable of generating positive DC voltage ranging from 10 to 30 kV, with 25 kV applied for this experiment.

A grounded rotating collector with a diameter of 45 mm was positioned 25 cm from the needle tip, and the rotation speed was maintained at 2000 rpm. The syringe containing the PHB solution was placed horizontally in a syringe pump (NE-300, New Era Pump Systems Inc.; Farmingdale, NY, USA) and delivered at a constant flow rate of 3 mL/h. Electrospinning was conducted for a deposition time of 6 h, at an ambient temperature of 25 °C, and a relative humidity of 51%. To eliminate any residual solvents, the electrospun PHB materials were dried in a heated vacuum desiccator (Vacuo-Temp, J.P. Selecta; Barcelona, Spain) at 30 °C.

### 2.3. Dip-Coating of the Electrospun PHB Materials

In order to study the film-forming ability on electrospun PHB materials, aqueous solutions of CMC-Na, HEC-L, HEC-M, and HEC-H at a concentration of 0.5 wt% were prepared. Additionally, a separate coating bath containing a *Bacillus subtilis* suspension at a concentration of 10% *v*/*v* relative to the cellulose derivatives was also prepared. In the first step of the dip-coating process, cut electrospun PHB samples (disks with a diameter of 16 mm) were immersed in the prepared aqueous solutions, both with and without *B. subtilis*, for 30 min. After immersion, the PHB mats were carefully withdrawn, blotted to remove excess liquid, and air-dried to a constant weight.

### 2.4. Characterization of the Obtained Electrospun Materials

Scanning electron microscopy (SEM) was used to conduct a complete morphological study of the prepared dip-coated electrospun PHB materials. All of the samples were vacuum-coated with gold for 60 s in a Jeol JFC-1200 fine coater before being observed on a Jeol JSM-5510 (JEOL Co., Ltd., Tokyo, Japan). Using Image J software, Version 1.54g at least 30 fibers from the SEM images were measured in order to determine the mean fiber diameter and the standard deviation. SEM analyses of the morphology of the *B. subtilis*-containing samples were performed after completely dried samples were vacuum-coated with gold for 60 s in a Jeol JFC-1200 fine coater and were observed on SEM Jeol JSM-5510 (JEOL Co., Ltd., Tokyo, Japan). The average number of *B. subtilis* cells was determined by counting the cells on area 14,000 μm^2^ by using five separate SEM micrographs captured on Jeol JSM-5510 (JEOL Co., Ltd., Tokyo, Japan).

The dynamic viscosities of the spinning solutions were measured on a Brookfield DV-II+ Pro programmable viscometer (Brookfield, Middleboro, MA, USA) equipped with a sample thermostatic cup and a cone spindle operated at 25 °C.

IRAffinity-1 spectrophotometer (Shimadzu Co., Kyoto, Japan) equipped with a MIRacle™ATR (diamond crystal, depth of penetration of the IR beam into the sample—about 2 μm) accessory (PIKE Technologies, Fitchburg, WI, USA) was used to record attenuated total reflection Fourier-transform infrared (ATR-FTIR) spectra of the obtained fibrous materials. The spectra were recorded in the range of 600–4000 cm^−1^ with a resolution of 4 cm^−1^. All spectra were corrected for H_2_O and CO_2_ using an IRsolution software program version 1.04.

Based on the surface wettability properties, the hydrophobic–hydrophilic behavior of the prepared dip-coated electrospun PHB materials was evaluated using an Easy Drop DSA20E Krűss GmbH apparatus (Hamburg, Germany). Computer analysis was used to calculate the average contact angle value after a sessile drop of deionized water (10 μL) was deposited onto the surface of the fibrous samples (2 cm × 7 cm cut in the direction of the collector rotation). Every sample was subjected to 10 measurements.

The tensile characteristics of the obtained dip-coated electrospun PHB samples were evaluated using a single-column system for mechanical testing INSTRON 3344, equipped with a loading cell 50 N and Bluehill universal software version 3.11. The strain rate was 10 mm/min, the initial length between the clamps was 40 mm and the room temperature was 21 °C. The width, length, and thickness of the tested specimens were 20 mm, 60 mm, and 400 μm, respectively. The average values of Young’s modulus (E, MPa), tensile strength (σ, MPa), and elongation at break (εB, %) were determined based on the regression of the linear part of the stress–strain curves from at least 10 tested specimens of each material.

### 2.5. Microbiological Tests

The viability of the beneficial bacteria encapsulated into electrospun dip-coated PHB materials was assessed by evaluating their ability to form colonies on solid TSA medium. Disks with a diameter of 16 mm, containing samples of the various biohybrid materials, were placed on the surface of sterilized TSA in Petri dishes and incubated at 28 °C. The formation and growth of bacterial colonies were monitored at 18, 48, and 72 h.

### 2.6. Statistical Data Analysis

In the present study, the results were reported as means ± standard deviation (SD). The statistical significance of the data was assessed using one-way analysis of variance (ANOVA), followed by a post hoc comparison test (Bonferroni) with the use of GraphPAD Prism software, version 5 (GraphPad Software Inc., San Diego, CA, USA). Statistically significant values were * *p* < 0.05, ** *p* < 0.01, and *** *p* < 0.001.

## 3. Results and Discussion

**Scheme 1:**
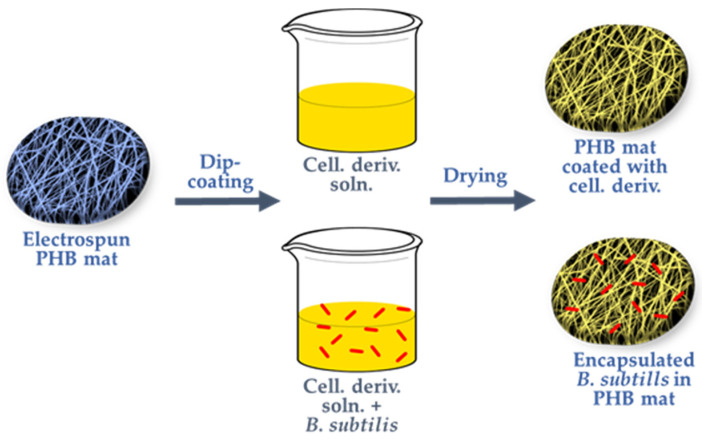
Schematic representation of the obtained PHB-based fibrous biohybrid materials.

Recently, we prepared electrospun poly(L-lactide) (PLLA) materials coated with chitosan as carriers for encapsulation of beneficial fungus *T. asperellum* and showed that these hybrid nanomaterials hamper the growth of the pathogenic *P. chlamydospora* and *P. aleophilum* fungi [22]. In this study, novel biohybrid materials were obtained as effective biocontrol formulations for plant protection and growth promotion, using the Gram-positive soil bacterium *Bacillus subtilis* as the biocontrol agent. Electrospun poly(3-hydroxybutyrate) (PHB) was selected as the polymer carrier due to its microbial origin, biodegradability, biocompatibility, and excellent mechanical properties, which are comparable to those of polypropylene [23]. In addition, a simple, low-cost, reliable, and reproducible dip-coating method was employed to form thin films on the electrospun PHB fibers (Scheme 1). This method preserves the desired fiber structure and facilitates the encapsulation of the beneficial bioagent. The dip-coating, utilizing water-soluble, biocompatible, and biodegradable natural cellulose derivatives, creates a favorable environment for the biocontrol agent, ensuring its survival during storage and normal development when introduced into an environment for plant growth or in the presence of moisture. Through this straightforward and innovative approach, the targeted biohybrid materials, based on dip-coated electrospun PHB fibers, were developed as effective biocontrol formulations.

The dip-coating method was chosen by considering several aspects of the technique that contribute to preserve the fibrous morphology of the electrospun PHB materials after the dip-coating process (Scheme 1). (i) Controlled application of the coating: In the dip-coating method, electrospun PHB materials are immersed in a solution with a controlled concentration of polymer or active agent. This ensures that the applied coating is thin and uniform, without filling or smoothing the fibrous structure. The thin coating leaves the fibers clearly defined and maintains their spatial organization. (ii) Solvent compatibility: The solvent used for the coating is selected so that it does not dissolve or deform the PHB fibers. The selected solvent does not degrade the material itself and the fiber morphology remains intact after application. (iii) Minimal physical intervention: During dipping, the materials are not subjected to mechanical pressure or temperature changes that could alter the fibrous structure. The dipping and drying process is gentle enough not to change the primary shape and arrangement of the fibers. (iv) Layered coating: The dip-coating can form a very thin layer over each fiber, rather than filling the spaces between them. In this way, the open porous structure of the electrospun fibers is preserved.

### 3.1. Morphological and Structural Characterization of Electrospun PHB Materials Coated with Cellulose Derivatives

Preliminary experiments were conducted to determine the optimal viscosity and concentration of the aqueous cellulose derivative solutions required to form a uniform thin film during dip-coating. The dynamic viscosities of the prepared aqueous solutions of CMC-Na, HEC-L, HEC-M, and HEC-H at a selected concentration of 0.5 wt% were subsequently measured. As anticipated, the solution viscosity increased with the molecular weight of the cellulose derivatives, following this order: HEC-L (10 cP) < CMC-Na (19 cP) ≈ HEC-M (20 cP) < HEC-H (38 cP).

**Figure 1:**
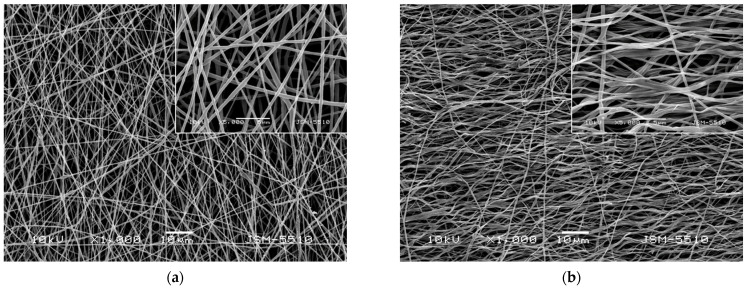

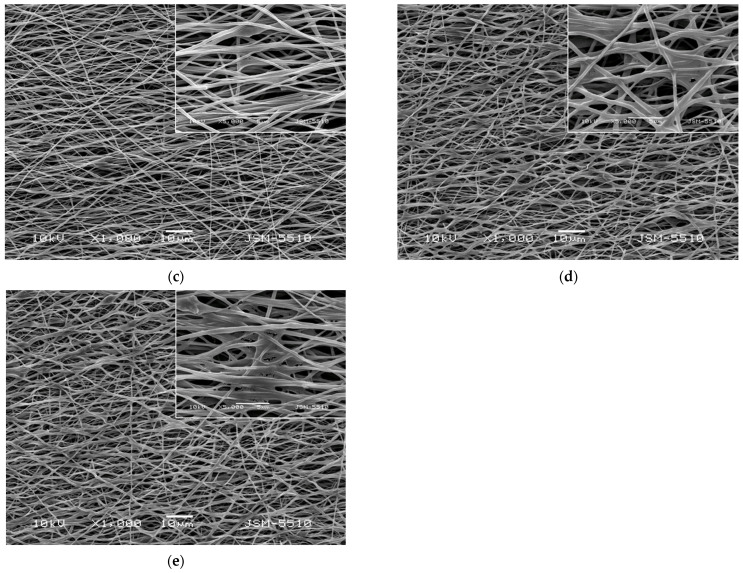
SEM images of the electrospun (**a**) PHB mat, (**b**) PHB mat coated with CMC-Na, (**c**) PHB mat coated with HEC-L, (**d**) PHB mat coated with HEC-M, and (**e**) PHB mat coated with HEC-H. Inset magnification ×5000.

The surface morphology of the obtained electrospun PHB materials, both before and after dip-coating in aqueous CMC-Na, HEC-L, HEC-M, and HEC-H solutions, was observed using SEM (Figure 1). The electrospinning of the PHB solution under the selected conditions prior to dip-coating resulted in the formation of uniform, defect-free cylindrical fibers without pores along their length (Figure 1a). Following dip-coating, the fiber structure remained intact, although some clumping of the fibers was observed (Figure 1b–e). This clumping is attributed to the deposition of a thin film of cellulose derivatives on the PHB fibers, which acts as an adhesive. The formation of this film over and between the PHB fibers is clearly visible in the SEM images (Figure 1b–e, insets). Additional evidence for the film formation is provided by the changes in mean fiber diameters measured before and after the dip-coating. Consistent with our previous findings, the mean PHB fiber diameter before dip-coating was 120 ± 22 nm [21]. After dip-coating with cellulose derivatives of varying molecular weights, the average diameters of the PHB fibers were as follows: 145 ± 25 nm for PHB/CMC-Na, 130 ± 23 nm for PHB/HEC-L, 150 ± 27 nm for PHB/HEC-M, and 165 ± 30 nm for PHB/HEC-H. The fiber diameter distribution was also determined and is presented in Figure S2. The increase in mean fiber diameter after coating is clearly due to the film deposition. As expected, the molecular weight of the cellulose derivatives also influenced the results, reflecting the different viscosities of the aqueous solutions.

**Figure 2:**
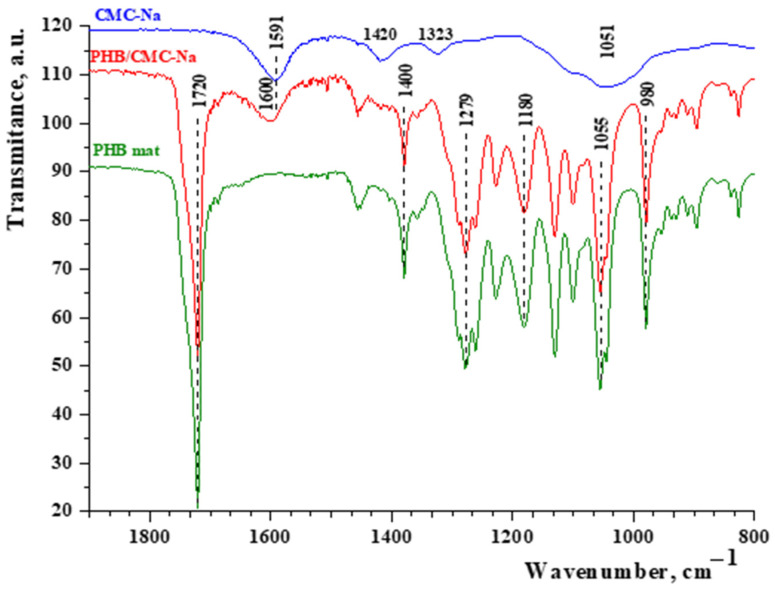
ATR-FTIR spectra of pristine CMC-Na (powder), electrospun PHB mat coated with CMC-Na, and electrospun PHB mat.

ATR-FTIR spectroscopy was used for qualitative analysis and to observe changes in the chemical structure of the electrospun PHB materials before and after dip-coating. The FTIR spectra of the electrospun PHB/CMC-Na materials were compared with those of pristine CMC-Na (powder) and electrospun PHB mats (Figure 2). The spectrum of pristine CMC-Na (powder) showed characteristic absorption bands at 1591 cm⁻^1^ and 1420 cm⁻^1^, corresponding to the asymmetric and symmetric stretching of –COONa. Additionally, an absorption band at 1325 cm⁻^1^, indicative of −OH bending vibrations, and a stretching band at 1051 cm⁻^1^ from the ether group (C–O–C) in the glucopyranose structure were also observed. In the representative spectrum of the PHB mat, the absorption peak at 1720 cm⁻^1^ corresponds to the C=O stretching band, while the peak at 1279 cm⁻^1^ is associated with –CH bending in the polymer chain. An absorption band at 1055 cm⁻^1^, attributed to C–O–C stretching, was also observed. In the FTIR spectrum of the PHB fibers coated with CMC-Na, the band at 1720 cm⁻^1^, characteristic of the C=O stretching in PHB, is still present. However, the characteristic carbonyl stretching band shifts to 1600 cm⁻^1^, compared to 1593 cm⁻^1^ in the CMC-Na spectrum. This shift indicates the formation of a thin CMC-Na film on the PHB fibers and suggests hydrogen bonding between functional groups of PHB and CMC-Na.

**Figure 3:**
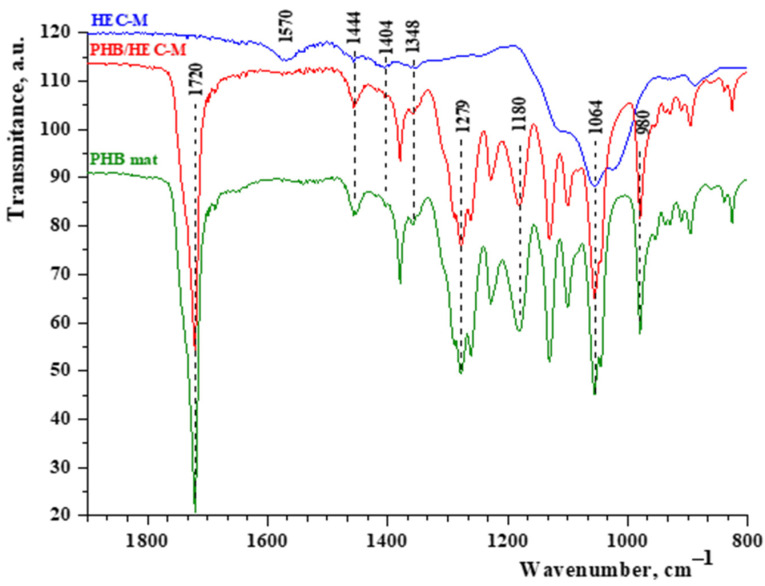
ATR-FTIR spectra of pristine HEC-M (powder), electrospun PHB mat coated with HEC-M, and electrospun PHB mat.

In addition, the FTIR spectrum of the electrospun PHB mat coated with HEC-M was compared to that of pristine HEC-M (powder) and electrospun PHB fibers (Figure 3). The characteristic absorption peaks for HEC at 1570 cm⁻^1^ and 1404 cm⁻^1^ are attributed to O–H plane deformation and C–H symmetric bending vibrations, respectively. A stretching band at 1064 cm⁻^1^, corresponding to the ether group (C–O–C) in the glucopyranose structure, was also observed. Moreover, a broad absorption peak at 3392 cm⁻^1^ due to O–H stretching vibrations and another peak at 2874 cm⁻^1^ from C–H stretching vibrations were detected (Figure S1). After coating the electrospun PHB fibers with HEC-M, characteristic peaks for both PHB and HEC were observed, indicating successful film formation. The characteristic PHB peak at 1720 cm⁻^1^, corresponding to the ester carbonyl group, and the peak at 1279 cm⁻^1^, associated with –CH bending in the polymer chain, were present. Simultaneously, HEC-specific peaks appeared, including a broad peak at 3392 cm⁻^1^, a peak at 2874 cm⁻^1^ (Figure S1), and the 1064 cm⁻^1^ peak assigned to C–O–C stretching in the glucopyranose structure of HEC (Figure 3). These FTIR analysis results confirm the successful coating of PHB fibers with thin films of cellulose derivatives.

The ability to design and control the surface wettability of the prepared fibrous PHB materials is crucial for their future applications. Morphological and structural analyses have demonstrated the successful coating of the PHB mats with cellulose derivative films, suggesting a change in their wettability. Specifically, a material surface that is easily wetted could either release or provide a conductive environment for encapsulated bioagents. To assess this, the water contact angle of the prepared PHB materials was measured, and the shape of the water droplet deposited on their surfaces was captured.

The molecular weight of the cellulose derivatives affects the contact angle by influencing surface energy, polymer chain orientation, and usually higher molecular weight derivatives leading to more hydrophobic surfaces. In high molecular weight materials, the cellulose chains may orient themselves in such a way that more hydrophobic functional groups are exposed on the surface, increasing the contact angle. Conversely, lower molecular weight derivatives might lead to a more hydrophilic surface due to less chain orientation, resulting in a lower contact angle and greater wettability.

**Figure 4:**
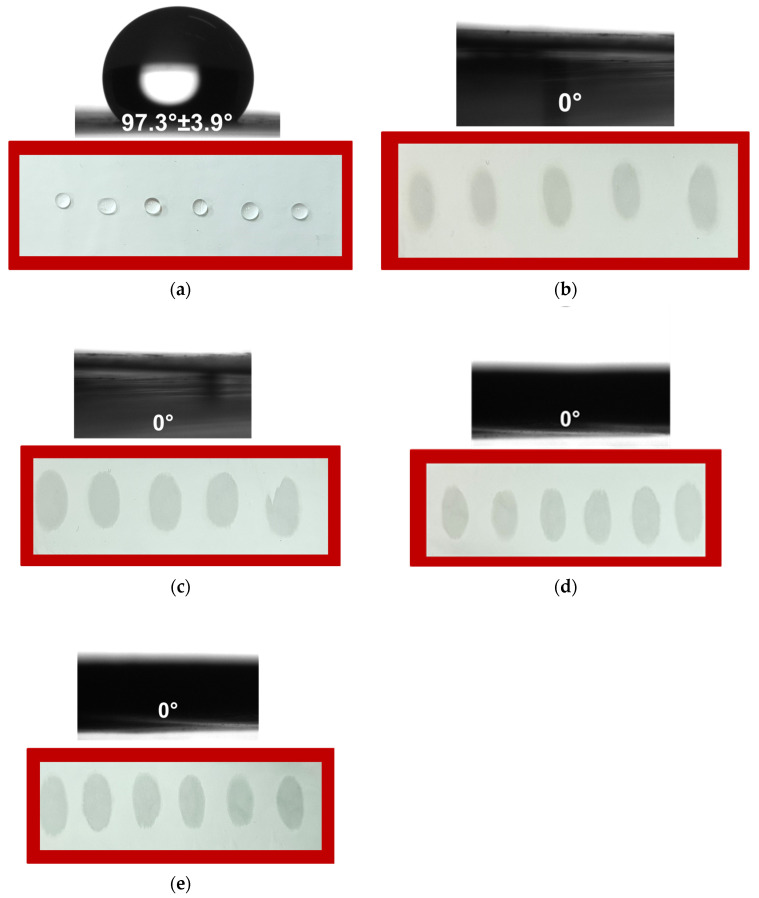
The water contact angle values and digital images of the droplets deposited on the electrospun: (**a**) PHB mat, (**b**) PHB mat coated with CMC-Na, (**c**) PHB mat coated with HEC-L, (**d**) PHB mat coated with HEC-M, and (**e**) PHB mat coated with HEC-H.

Digital images of water droplets on the surface of the PHB mat, as well as those coated with different cellulose derivatives, along with the corresponding contact angle values, are shown in Figure 4. As observed, the surface of the electrospun PHB mat is hydrophobic, with a water contact angle of approximately 97°. The water droplets on the PHB mat surface are round and do not spread (Figure 4a). In contrast, the electrospun PHB mats coated with cellulose derivatives are hydrophilic, with a water contact angle of 0° (Figure 4b–e). The significant decrease in water contact angle is attributed to the film-forming of CMC-Na and HEC onto PHB mats. In addition, the contact angle for all coated fibrous mats was found to be independent of the molecular weight of the cellulose derivative used. The water droplets were absorbed immediately upon contact with the coated PHB surface, indicating that PHB mats coated with CMC or HEC exhibit superhydrophilic properties.

### 3.2. Effect of a Cellulose Derivative Coating on the Mechanical Properties of Electrospun PHB Materials

The effect of a cellulose derivative coating on the mechanical properties of electrospun PHB materials was investigated through tensile tests. Recently, we have demonstrated that electrospun PHB samples cut in the direction of collector rotation (0°) exhibited superior mechanical properties [24]. Therefore, in this study, all PHB specimens were cut in the direction of collector rotation.

The molecular weight and viscosity of cellulose derivatives play key roles in determining the mechanical properties of the material, where higher molecular weight and viscosity lead to stronger, more resilient structures. Viscosity is influenced by molecular weight—higher molecular weight derivatives typically form more viscous solutions. In coating process, high-viscosity solutions can lead to thicker fibers or coatings, which may enhance tensile strength and elasticity. Conversely, lower viscosity solutions can result in thinner fibers with less mechanical resilience.

**Figure 5:**
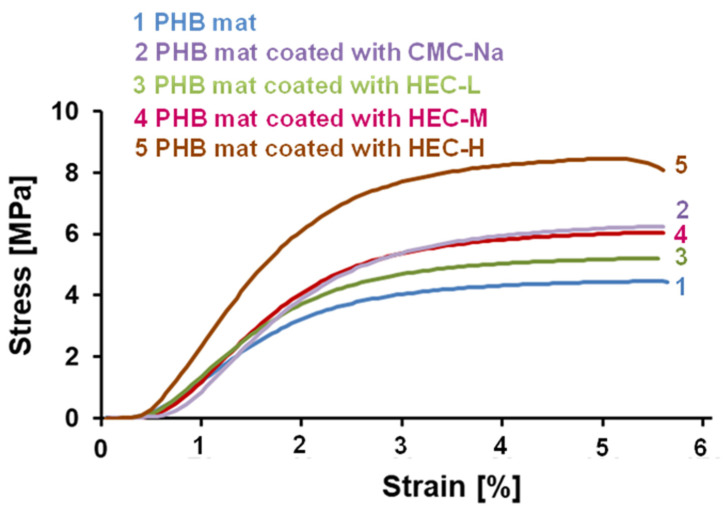
Stress–strain curves of electrospun (1) PHB mat, (2) PHB mat coated with CMC-Na, (3) PHB mat coated with HEC-L, (4) PHB mat coated with HEC-M, and (5) PHB mat coated with HEC-H.

The resultant stress–strain curves of the electrospun PHB materials before and after coating with cellulose derivatives are shown in Figure 5. The uncoated electrospun PHB mat exhibited a tensile strength of 4.45 MPa, consistent with previously reported values [21]. The coating process significantly enhanced the mechanical properties of the electrospun PHB materials, as indicated by curves 2, 3, 4, and 5 in Figure 5. The molecular weight of the cellulose derivatives, specifically CMC-Na and HEC, had a substantial impact on the mechanical characteristics of the electrospun PHB materials. As the molecular weight of the cellulose derivative increased, so did the tensile strength, following the order: HEC-L (5.2 MPa) < CMC-Na (6.24 MPa) ≈ HEC-M (6.23 MPa) < HEC-H (8.4 MPa). Notably, coating the electrospun PHB materials with high molecular weight HEC resulted in a twofold increase in tensile strength compared to the uncoated PHB mat. Thus, the dip-coating of electrospun materials, followed by film formation, proves to be an effective approach for significantly improving the mechanical properties of electrospun materials.

### 3.3. Morphological Characterization of Electrospun Biohybrid Materials

*Bacillus subtilis* is a Gram-positive, catalase-positive bacterium with a rod-shaped structure capable of forming tough, protective endospores, which allow it to withstand extreme environmental conditions. It is one of the most efficient bacteria for producing secreted enzymes, making it widely used by biotechnology companies on a large scale. Before the advent of antibiotics, *B. subtilis* cultures were popular in alternative medicine as an immunostimulatory agent, aiding in the treatment of gastrointestinal and urinary tract diseases. In agriculture, this bacterium is employed as a soil inoculant and has been found to be an effective bioproduct fungicide. Given these benefits, and with the aim of fabricating effective biocontrol formulations for sustainable agricultural applications, this study presents the first successful encapsulation of *B. subtilis* into electrospun PHB materials coated with cellulose derivatives. The dip-coating method used preserves the desired fibrous structure of the polymer carrier, enhances mechanical properties, and facilitates the encapsulation of this beneficial microorganism.

Additionally, the applied cellulose derivative coating provides a viable environment for the encapsulated bioagent during storage and transportation, ensures its survival in the target environment, and supports its normal development in the presence of moisture. Schematic representation of the encapsulation of *Bacillus subtilis* in cellulose derivative coatings onto PHB mats is shown in Scheme 1.

The distribution and concentration of *Bacillus subtilis* cells on PHB mats coated with different cellulose derivatives are driven by multiple factors, including the viscosity and molecular weight of the coating solution, the adhesion properties of the derivative, surface morphology, nutrient diffusion, and interactions between the coating and the PHB substrate. The molecular weight and viscosity of cellulose derivatives contribute to a more controlled and uniform distribution of *B. subtilis* cells by limiting cell mobility during encapsulation. High-viscosity solutions provide a more uniform environment for the distribution of *B. subtilis* cells within the electrospun PHB material. The increased viscosity reduces the mobility of the cells during encapsulation, leading to a more controlled and even distribution. In contrast, low-viscosity solutions may allow cells to move freely before the material sets, potentially leading to uneven distribution or clustering.

**Figure 6:**
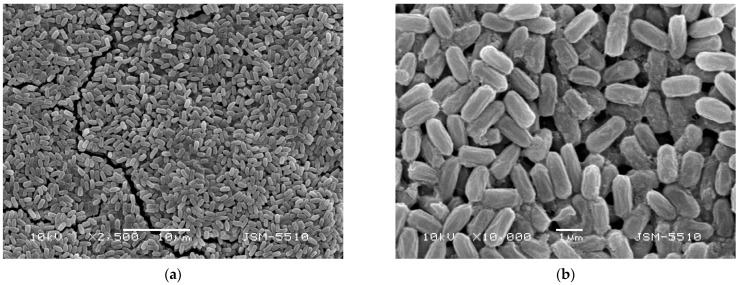

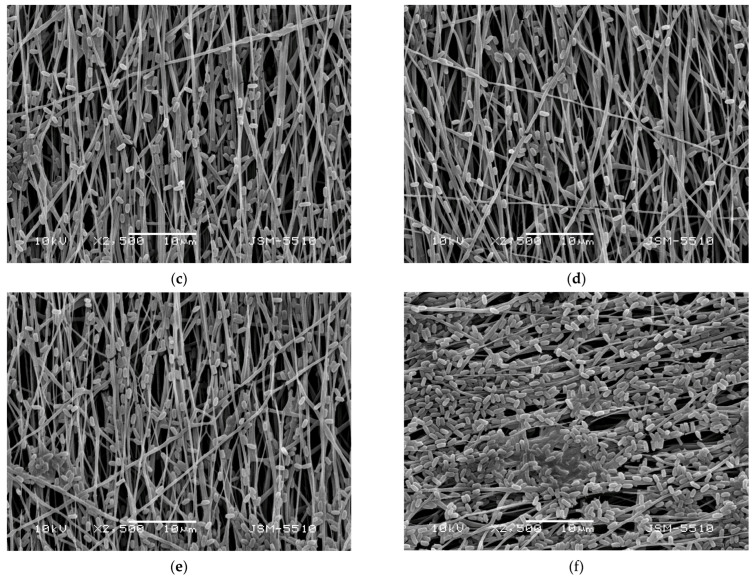
SEM images of: (**a**) *B. subtilis* ×2500; (**b**) *B. subtilis* ×10,000; (**c**) PHB mat coated with CMC-Na/*B. subtilis*; (**d**) PHB mat coated with HEC-L/*B. subtilis*, (**e**) PHB mat coated with HEC-M/*B. subtilis*; and (**f**) PHB mat coated with HEC-H/*B. subtilis*. Magnification ×2500.

The morphology of the *B. subtilis* cultures was observed using SEM (Figure 6a,b). The bacterial spores are rod-shaped, measuring 1.474 ± 0.164 µm in length and 0.729 ± 0.887 µm in diameter. As mentioned, after dip-coating the electrospun PHB materials with aqueous cellulose derivative solutions containing a suspension of *B. subtilis*, the appearance of spores on the PHB surface was observed (Figure 6c–f). The number of cells determined from the SEM micrographs per 14,000 μm^2^ on the PHB mats coated with cellulose derivatives was approximately 470 cells on the PHB mat coated with CMC-Na/*B. subtilis* and 350, 520, and 1185 cells on PHB mats coated with HEC-L/B. subtilis, HEC-M/*B. subtilis*, and HEC-H/*B. subtilis*, respectively. It is evident that coating with CMC-Na, HEC-L, and HEC-M results in a uniform distribution of cells on the PHB fibers. As the molecular weight increases, besides an overall increase in cell distribution, there are zones with higher concentrations where the polysaccharide film is thicker. The PHB mat saturated with the most *B. subtilis* bacteria is the material coated with HEC-H, where the molecular weight and viscosity of the corresponding solution are the highest. In this case, a very dense, uniform film consisting of bacterial cells is formed (Figure 6f). Nevertheless, *B. subtilis* is visibly present in all fibrous PHB materials coated with cellulose derivative films.

### 3.4. Viability of B. subtilis in Electrospun Biohybrid Materials

After successfully encapsulating *Bacillus subtilis* in electrospun PHB materials, it was crucial to verify whether the bacterial cells remained viable and could grow normally. To assess this, cell viability tests were conducted by placing the PHB mats coated with cellulose derivatives/*B. subtilis* films on agar plates.

The multi-timepoint approach was used to ensure a comprehensive assessment of the growth dynamics and viability of *B. subtilis* within the biohybrid material, covering the entire bacterial growth cycle from early division to long-term survival and adaptation. Early growth-phase measurements at 18 h capture early bacterial growth and adaptation to the biohybrid material. The timepoint at 48 h is a critical phase for understanding bacterial–material interactions, assessing bacterial viability and growth, and providing insights into how well the biohybrid material is supporting sustained bacterial activity over time. The 72 h timepoint is crucial for assessing the long-term compatibility of the biohybrid material with bacterial cells and provides insights into its long-term viability and survival.

**Figure 7:**
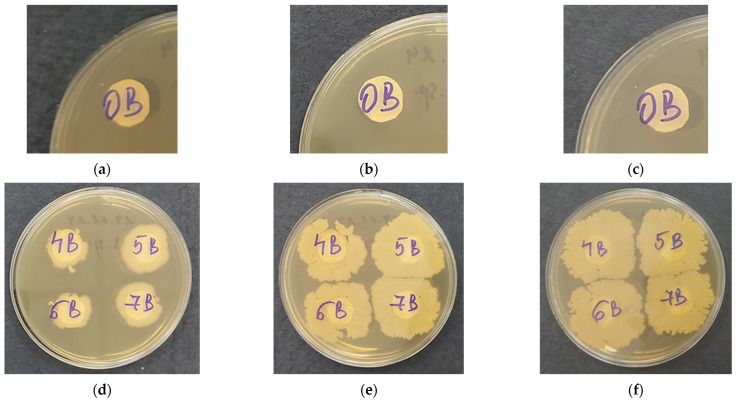
Digital images of the electrospun mats after (**a**,**d**) 18 h, (**b**,**e**) 48 h, and (**c**,**f**) 72 h of incubation: (**a**–**c**) 0B—PHB mat (control); (**d**–**f**) 4B—PHB mat coated with CMC-Na/*B. subtilis*; 5B—PHB mat coated with HEC-L/*B. subtilis*; 6B—PHB mat coated with HEC-M/*B. subtilis* and 7B—PHB mat coated with HEC-H/*B. subtilis*.

Digital images of *B. subtilis* growth at 18, 48, and 72 h of incubation are shown in Figure 7. As expected, no growth was observed around the control electrospun PHB mat. After 18 h, initial growth was detected around the samples coated with cellulose derivatives/*B. subtilis*, with slightly less growth observed around the electrospun PHB mat coated with CMC-Na/*B. subtilis*. By 48 h, *B. subtilis* continued to grow in all samples, and by 72 h, the bacterial cells had spread across the entire Petri dish. These results confirm that the bacterial cells remain viable within the cellulose derivative films and are capable of normal growth from the hybrid materials.

## 4. Conclusions

Eco-friendly biohybrid materials were successfully obtained using a simple and effective approach that combines the advantageous properties of electrospun PHB materials, cellulose derivatives, and the biocontrol agent *Bacillus subtilis*. For the first time, this biocontrol agent was encapsulated in electrospun PHB fibers by dip-coating. Morphological studies revealed that the bacterial cells were uniformly distributed throughout the biohybrid material. The cellulose derivative coating acted as an adhesive for the PHB fibers and played a crucial role in maintaining the viability of the encapsulated beneficial bacteria.

The study assessed the impact of cellulose derivative molecular weight and viscosity on the morphological, mechanical, and biological properties of the prepared fibrous materials. Coating the PHB fibers enhanced their mechanical properties compared to uncoated PHB fibers, with the highest tensile strength (8.4 MPa) observed in PHB mats coated with HEC-H. Microbiological tests confirmed that bacterial cells embedded in electrospun PHB mats coated with CMC-Na or HEC remained viable and grew normally. After 72 h of incubation, bacterial growth from the hybrid materials covered the entire Petri dish. These novel biocontrol formulations hold significant potential for sustainable eco-agriculture, providing effective solutions for plant protection and growth promotion.

## Data Availability

Data are contained within the article.

## Supplementary Materials

The following supporting information can be downloaded at: https://www.mdpi.com/article/10.3390/polym16192749/s1, Figure S1: ATR-FTIR spectra of pristine HEC-M (powder), electrospun PHB mat coated with HEC-M and electrospun PHB mat in the range of 2600 to 3600 cm^−1^. Figure S2: Diameter distribution of the fibers: (a) PHB mat, (b) PHB mat coated with CMC-Na, (c) PHB mat coated with HEC-L, (d) PHB mat coated with HEC-M, and (e) PHB mat coated with HEC-H.
