# Supplementary material for: Encapsulation of Bacillus subtilis in Electrospun Poly(3-hydroxybutyrate) Fibers Coated with Cellulose Derivatives for Sustainable Agricultural Applications
Source: Polymers (Basel). 2024 Sep 28;16(19):2749. doi: 10.3390/polym16192749 (PMC11479013; doi:10.3390/polym16192749)
Supplement: Supplementary file 1 [file polymers-16-02749-s001.zip › polymers-3222904-supplementary.pdf]

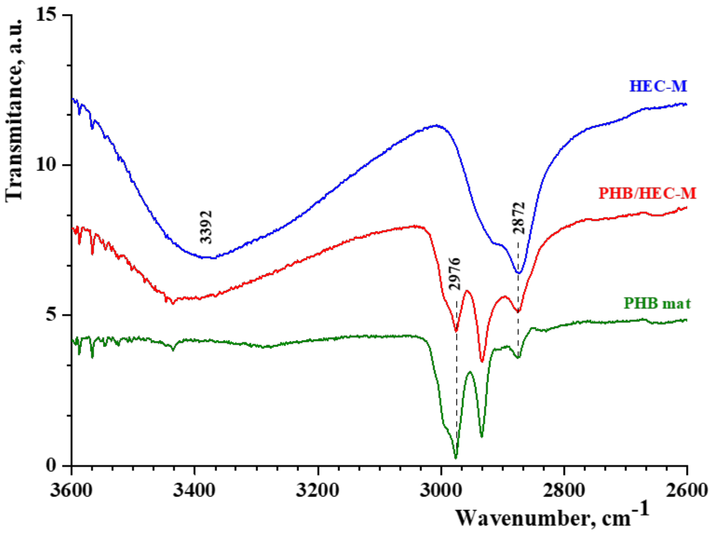

Figure S1. ATR-FTIR spectra of pristine HEC-M (powder), electrospun PHB mat coated with HEC-M and electrospun PHB mat in the range of 2600 to 3600 cm^{-1}.

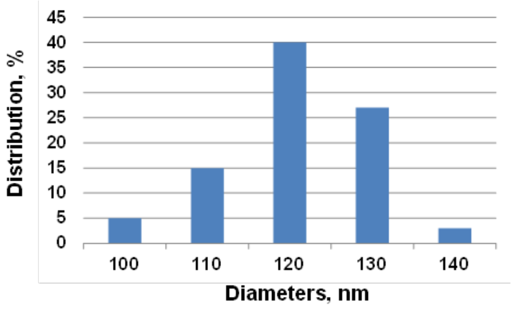

(a)

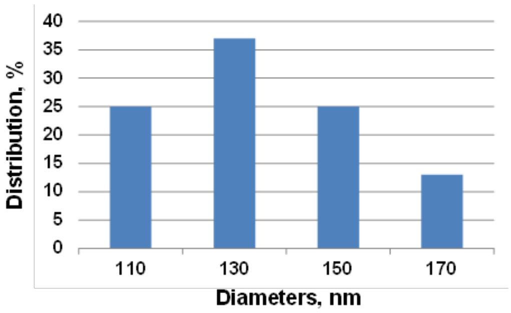

(b)

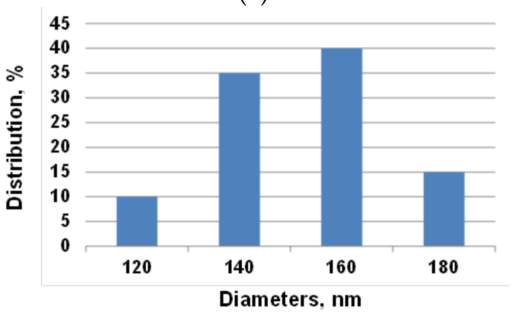

(c)

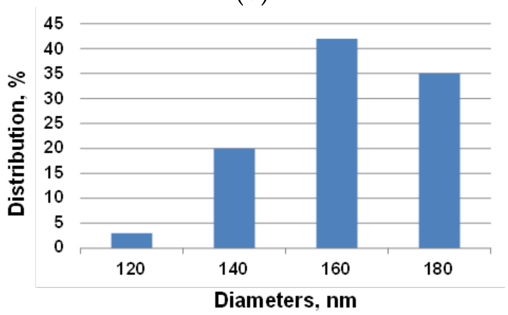

(d)

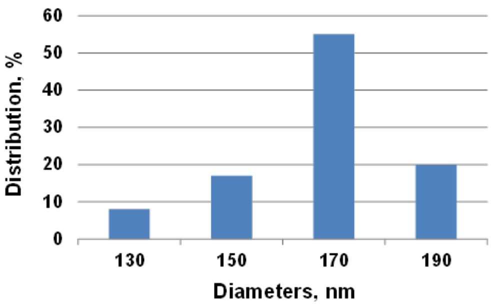

(e)

Figure S2. Diameter distribution of the fibers: (a) PHB mat, (b) PHB mat coated with CMC-Na, (c) PHB mat coated with HEC-L, (d) PHB mat coated with HEC-M and (e) PHB mat coated with HEC-H.
